# Verrucous Carcinoma of Tongue in Xeroderma Pigmentosum: A Case Report and Literature Review

**DOI:** 10.7759/cureus.31706

**Published:** 2022-11-20

**Authors:** Arens Jean Ricardo Medeus, Ansly Jefferson Desravines, Vivianne Cotard

**Affiliations:** 1 Surgery, Hôpital de l'Université d'Etat d'Haïti, Port-au-Prince, HTI; 2 Dermatology, Hôpital de l'Université d'Etat d'Haïti, Port-au-Prince, HTI

**Keywords:** ophthalmology, dermatology, squamous cell carcinoma, verrucous carcinoma, xeroderma pigmentosum

## Abstract

Xeroderma pigmentosum (XP) is a rare autosomal recessive genetic disorder characterized by intense skin photosensitivity that is often associated with corneal ulceration, erythema, malignant lesions in sun-exposed areas, and neurological damage in severe cases. XP is due to alterations in the nucleotide excision repair system which could eliminate DNA fragments damaged by ultraviolet radiation.

We report a case of a 14-year-old admitted for photophobia and a conjunctival mass. He underwent laboratory tests, including a complete blood count (CBC), which was unremarkable, and serological tests such as rapid plasma reagin (RPR) and human immunodeficiency virus (HIV) test were negative. A consultation in Ophthalmology was requested, concluding in bilateral corneal dystrophy. A few months later he developed two masses, one on the distal border of the tongue and the other at the level of the parotid region. He underwent two excisional biopsies; the parotid mass revealed an ulcerated squamous cell carcinoma on a background of xeroderma pigmentosum, and the tongue tip mass revealed a well-differentiated infiltrating verrucous carcinoma with a smooth margin.

Xeroderma pigmentosum is a rare genodermatosis affecting the skin, eyes and oral cavity. It is sometimes associated with cancers of internal organs and rarely of the tongue. This study reports a case of XP associated with verrucous carcinoma of the tongue and ocular complications. Currently, there is no curative treatment for XP, and the only treatments available are symptomatic and preventive.

## Introduction

Xeroderma pigmentosum (XP) is a rare autosomal recessive genetic disorder with a prevalence of 1 to 4 cases per 300,000 population in Europe and the United States, originally described by Moriz Kaposi in 1874 [1]. XP is clinically characterized by intense cutaneous photosensitivity that is often associated with corneal ulceration, erythema, malignant lesions in sun-exposed areas, and neurological involvement in severe cases [2]. It is caused by alterations in the nucleotide excision repair system, which could remove deoxyribonucleic acid (DNA) fragments damaged by ultraviolet radiation [3]. Because of this hypersensitivity to ultraviolet radiation, people with this condition have a high risk of skin cancer. XP can be classified into eight types: XP-A to XP-G and XP-V, depending on the gene affected by the mutation [3].

In Haiti, dermatology is a marginalized specialty; thus, many skin disorders may be underdiagnosed due to the limitations of diagnostic equipment and the economic means of patients, and there are very few studies in this field in the country. We present the case of a 14-year-old male adolescent with xeroderma pigmentosum. This study is the first case reported in Haiti and one of the rare cases of xeroderma pigmentosum described in the literature associated with verrucous tongue carcinoma.

## Case presentation

**Figure 1:**
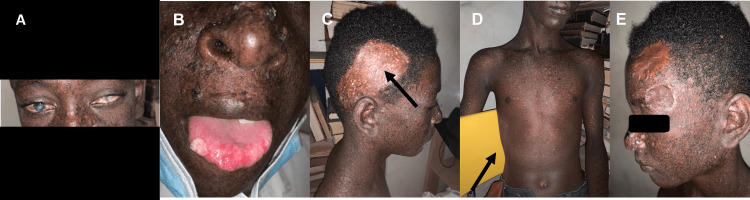
(A) Dystrophy corneal; (B) Verrucous carcinoma on the tongue tip; (C) Alopecic plaque, an ulcerated oozing mass alternated with small masses on the scalp; (D) Hyper and hypochromic macules disseminated on the whole body; (E) Actinic keratosis lesions.

This is a 14-year-old boy, followed in the Dermatology department for 10 years, with no family history of consanguinity. He was admitted for photophobia and conjunctival mass; he underwent laboratory tests, including a complete blood count (CBC), which was unremarkable, and serological tests such as rapid plasma reagin (RPR) and human immunodeficiency virus (HIV) test were negative. A consultation in Ophthalmology was requested concluding in bilateral corneal dystrophy (Figure 1A); a few months later he developed two masses one at the level of the tip of the tongue (Figure 1B) and the other at the level of the parotid region. He underwent two excisional biopsies; the parotid mass revealed an ulcerated squamous cell carcinoma, and the tongue tip mass revealed a well-differentiated infiltrating verrucous carcinoma (Figure 1B) with a smooth margin. He developed an alopecic plaque, an ulcerated oozing mass alternated with small masses on the scalp (Figure 1C), and presented hyperpigmented and hypopigmented macules widely disseminated (Figure 1D). At the same time, actinic keratosis lesions appeared on his face and upper limbs, for which imiquimod was applied (Figure 1A, Figure 1E). Small tumors have also been detected on the nose and cheekbones, as well as budding lesions that bled at the slightest contact on the lower lip. He was placed on sunscreen 50 +, morning and noon application, Heliocare one tablet per day, 5-fluorouracil cream one application each evening, and cephalexin one tablet twice a day for 10 days. Another ophthalmological examination was requested, revealing an oozing mass at the level of the left eyebrow arch, opacification of both corneas, and degenerative keratopathy of the lipid type, so he was placed on bacitracin and polymyxin B ophthalmic ointment twice per day, prednisone acetate eye drops six times a day, sodium chondroitin sulfate/sodium hyaluronate eye drops twice per day and encouraged to wear sunglasses.

The patient had good evolution and was placed on local care with trichloroacetic acid for touching the budding lesions every morning, betadine for cleaning the oozing lesion morning and evening, and sodium fusidate cream morning and evening on the ulcerated lesion.

## Discussion

Xeroderma pigmentosum (XP) is a rare genodermatosis in which patients have extreme sensitivity to ultraviolet radiation; although there is heterogeneity in the presentation of symptoms, patients most often present with lesions in exposed areas such as the upper limbs, neck, and face; in addition, patients may present neurological and ophthalmological abnormalities [1,4]. Among the eight genes identified as responsible for XP (XP-A to XP-G and XP-V), studies have shown that the first seven subtypes have a mutation in nucleotide excision repair. In the last type, XP-V, the patient has a functional nucleotide excision repair system, but the problem is in the post-replication repair [3,4].

At this time, there is no curative treatment for XP; treatments are symptomatic and preventive such as using sunscreens, vitamins, and retinoids. There is also preventive chemotherapy with fluorouracil and topical imiquimod. Early removal of precancerous lesions is also advocated to avoid the occurrence of cancer [5]. Unfortunately, patients with the latter complication have an average lifespan of no more than 37-39 years [6].

Cancer of internal organs such as lung, blood or colon in patients with XP has been reported in some studies [7-10], but there are very few cases of cancer developed in the tongue that have been reported in the literature [10-12]. In this study, we report a verrucous carcinoma which is a well-differentiated squamous cell carcinoma with a low degree of malignancy. This is the first case of xeroderma pigmentosum described in Haiti and one of the few cases of xeroderma described in the literature associated with a verrucous carcinoma of the tongue.

## Conclusions

Xeroderma pigmentosum is a rare genodermatosis affecting the skin, eyes and oral cavity. It is sometimes associated with cancers of internal organs and rarely of the tongue. This study reports a case of XP associated with verrucous carcinoma of the tongue and ocular complications. Currently, there is no curative treatment for XP, and the only treatments available are symptomatic and preventive.
